# Early use of alendronate as a protective factor against the development of glucocorticoid-induced bone loss in childhood-onset rheumatic diseases: a cross-sectional study

**DOI:** 10.1186/s12969-018-0258-5

**Published:** 2018-06-18

**Authors:** Yuzaburo Inoue, Kanako Mitsunaga, Takeshi Yamamoto, Koki Chiba, Fumiya Yamaide, Taiji Nakano, Yoshinori Morita, Akiko Yamaide, Shuichi Suzuki, Takayasu Arima, Ken-ichi Yamaguchi, Minako Tomiita, Naoki Shimojo, Yoichi Kohno

**Affiliations:** 1 Department of Pediatrics, Eastern Chiba Medical Center, 3-6-2 Okayamadai, Togane, Chiba, 283-8686 Japan; 2 Department of General Medical Science, Graduate School of Medicine, Chiba University, Chiba, Chiba Japan; 3 Department of Pediatrics, Chiba Kaihin Municipal Hospital, Chiba, Chiba Japan; 4 Department of Allergy and Rheumatology, Chiba Children’s Hospital, Chiba, Chiba Japan; 5 Department of Traditional Medicine, Toho University Omori Medical Center, Ota-ku, Tokyo, Japan; 6 Department of Pediatrics, Graduate School of Medicine, Chiba University, Chiba, Chiba Japan; 7 Department of Pediatrics, IMS Memorial Hospital, Itabashi-ku, Tokyo, Japan; 8 Department of Pediatrics, Shimoshizu National Hospital, Yotsukaido, Chiba, Japan; 9 Department of Pediatrics, Kimitsu Chuo Hospital, Kisarazu, Chiba, Japan; 10 Immuno-Rheumatology Center, St.Luke’s Internationl Hospital, Chuou-ku, Tokyo, Japan; 11 Chiba Rosai Hospital, Ichihara, Chiba, Japan

**Keywords:** Osteoporosis, Bone loss, Alendronate, Glucocorticoid, Childhood-onset rheumatic disease

## Abstract

**Background:**

Bisphosphonates are recommended for use as first-line therapy for the prevention and treatment of glucocorticoid-induced osteoporosis in adults. However, the appropriate usage of bisphosphonates for the prevention or treatment of glucocorticoid-induced osteoporosis in children remains unclear.

**Methods:**

We performed a cross-sectional study to clarify the factors associated with the development of glucocorticoid-induced bone loss and osteoporosis in patients with childhood-onset rheumatic disease and to investigate the impact of the early use of alendronate. We recruited 39 patients with childhood-onset rheumatic disease who were evaluated to detect bone loss or osteoporosis at 3 months to 1.5 years after the initiation of treatment. The primary outcome of the study was the presence of bone loss or osteoporosis at the initial evaluation of the bone mineral density after at least 3 months of glucocorticoid therapy.

**Results:**

Bone loss and a history of fracture were found in 56 and 18% of the participants, respectively. Weekly oral alendronate therapy (median, 25.4 mg/m^2^) had been started by the time of the evaluation of osteoporosis in 46% of the participants and within 3 months after the start of glucocorticoid in 31% of the participants. There were no significant differences between the participants with bone loss (wBL group) and without bone loss (w/oBL group) in terms of gender, primary disease, or the age at the onset of primary disease. In terms of glucocorticoid use, there was no significant difference in the age at the start of glucocorticoid therapy, the length of glucocorticoid use, or the dose of glucocorticoids. The proportion of patients in the w/oBL group who received alendronate within 3 months after the start of glucocorticoid therapy was significantly greater than that in the wBL group. In the logistic regression analysis, only “alendronate therapy within 3 months after the start of glucocorticoid therapy” had a statistically significant effect on the development of bone loss (OR, 0.08; 95% CI, 0.02–0.43). The analysis did not reveal any factors associated with the development of osteoporosis.

**Conclusions:**

The early use of alendronate may have a preventive effect against the development of bone loss in glucocorticoid-treated patients with childhood-onset rheumatic disease.

**Electronic supplementary material:**

The online version of this article (10.1186/s12969-018-0258-5) contains supplementary material, which is available to authorized users.

## Background

Osteoporosis, one of the most serious adverse effects of glucocorticoid treatment, can occur in both adults and children [1]. In addition to having an increased risk of fracture [2], children receiving glucocorticoid treatment are at risk of developing a low peak bone mass [3], which may be associated with a long-term risk of fracture, as bone loss occurs during the period in which children should reach a normal peak bone mass.

It has been shown that bone loss and an increased fracture rate occur early after the initiation of glucocorticoid therapy [4], due to relatively higher bone resorption and suppressed bone formation, which occur due to glucocorticoid treatment. Thus, the guidelines for glucocorticoid-induced osteoporosis in adults recommend the prevention of osteoporosis, rather than treatment, recommended for all patients for whom long-term glucocorticoids is planned [5]. Bisphosphonates are synthetic derivatives of pyrophosphonates, which inhibit bone resorption—due to their action on osteoclasts [6]—and increase the bone mineral density (BMD), and are thus recommended for use as first-line therapy for the prevention and treatment of glucocorticoid-induced osteoporosis in adults [5]. However, the appropriate usage of bisphosphonates for the prevention or treatment of glucocorticoid-induced osteoporosis in children remains unclear.

We previously found that the efficacy of alendronate, a bisphosphonate, in the treatment of glucocorticoid-induced osteoporosis in children significantly correlated with a high level of bone turnover markers before alendronate treatment [7], suggesting that early intervention with bisphosphonates during periods of high bone turnover might be effective for both the treatment and prevention of glucocorticoid-induced osteoporosis.

We therefore performed a cross-sectional study to clarify the factors associated with the development of glucocorticoid-induced bone loss and osteoporosis in patients with childhood-onset rheumatic disease and to investigate the impact of the early use of alendronate.

## Methods

### Study design

We performed a cross-sectional study of patients with childhood-onset rheumatic disease who received glucocorticoid therapy. The study was performed according to the principles of the Declaration of Helsinki. The study protocol was approved by the Ethical Review Board of Chiba University Hospital (Approval number, 2887). Written informed consent was obtained from each of the study participants and their guardians.

### Setting and participants

The study recruited patients who had childhood-onset rheumatic disease and who were treated with glucocorticoid in Chiba University Hospital, which is located in an urban area in Japan, from October to November 2017. Chiba University Hospital is thought to serve approximately half of the pediatric patients with rheumatic disease in the area due to the small number of centers treating pediatric rheumatic diseases. The eligible participants were patients who started glucocorticoid therapy at Chiba University Hospital between 1984 and 2014, who were < 16 years of age, and who were diagnosed with either bone loss or osteoporosis after at least 3 months of treatment. The exclusion criteria were a lack of medical records, a past history of bone disease, metabolic disease, or other factors affecting the BMD. Patients in whom the initial evaluation of BMD was performed after 1.5 years of the treatment were also excluded from the study. We confirmed the diagnoses of the participants using the American College of Rheumatology diagnostic criteria.

### Data collection

- The diagnosis of bone loss and osteoporosis

The primary outcome of the study was the presence of bone loss or osteoporosis at the initial evaluation of the bone mineral density after at least 3 months of glucocorticoid therapy. The BMD of the spine L2–4 (LS BMD, anterior-posterior view) was measured using a dual X-ray absorptiometry device (QDR 1000 plus or QDR Discovery, Hologic, MA, USA). To evaluate the Z-score, we used the standard LS BMD for healthy Japanese children and adolescents [8]. Bone loss was diagnosed when the Z-score of the LS BMD was ≤ − 2. As glucocorticoid-induced osteoporosis in childhood had not been defined due to insufficient evidence of risk factors for fragility fractures in the population, we used the definition of osteoporosis in children and adolescent recommended by the International society for clinical densitometry [9]. The diagnosis was based on the presence of both a clinically significant fracture history and a low BMD. A clinically significant fracture history is defined by the presence of one or more of the following factors: long bone fracture of the lower extremities, vertebral compression fracture, two or more long bone fractures of the upper extremities. In all cases, a semi-quantitative method was used to evaluate plain radiography of the thoracolumbar spine to identify fractures and to diagnose any asymptomatic fractures [10].

- Use of glucocorticoids

The length and dose of glucocorticoid use were checked from the medical records. The cumulative dose of glucocorticoids was calculated based on the prednisolone-equivalent anti-inflammatory effects. As the effect of methylprednisolone pulse therapy on the development of bone loss was considered to be independent from that of daily oral glucocorticoid use [11], we counted the number of times methylprednisolone pulse therapy was administered. In addition, we evaluated the cumulative prednisolone-equivalent dose, including or excluding the glucocorticoid dose associated with methylprednisolone pulse therapy.

- Supplementation of vitamin D and calcium

To avoid the development of urinary stones, we prescribed of vitamin D or calcium supplementation to the participants whose urinary calcium-to-creatinine ratio had been < 0.2 for at least 2 months. These patients took 400 IU of vitamin D daily and/or 1200 mg of calcium carbonate.

- Alendronate therapy

Based on our hospital’s experience with the use of bisphosphonates and previous reports, we recently used oral alendronate for patients with childhood-onset rheumatic diseases who were expected to receive glucocorticoid at a dose of ≥0.1 mg/kg/day for 3 months or longer. As shown in our previous paper [7], the effects of alendronate depend on the bone turnover of patients before treatment. Thus, we treated the patients with oral alendronate as early as possible, even if they did not have fragility fractures at that time. Written informed consent for the preventive usage of alendronate was obtained from each patient and their guardians. The dose of oral alendronate was determined based on the low oral bioavailability (< 1%) of alendronate [12]. The patients were treated with oral alendronate weekly (35 mg for ≥30 kg, 25 mg for 20 kg to < 30 kg, 15 mg for 15 kg to < 20 kg). They were advised to take the study medication on an empty stomach 30–60 min before breakfast, with water, and to avoid lying down for 1 h after taking the medication.

### Statistical methods

Non-Gaussian distributed variables were described as the median and interquartile range. The Mann-Whitney test and Fisher’s exact probability test were used to analyze the differences in the characteristics and outcomes between participants with and without bone loss. The odds ratio and confidence intervals were also calculated. In cases in which a value of zero caused problems with the calculation of the odds ratio, 0.5 was added to all of the cells and the adjusted odds ratio was calculated. A stepwise multivariate logistic regression analysis was performed to assess the relationship between the presence of bone loss and osteoporosis. We estimated the individual odds ratios and their confidence intervals.

In all of the analyses, *p* values of < 0.05 were considered to indicate statistical significance. All statistical analyses were performed using the JMP® Pro 12 (SAS Institute Inc., NC) and Prism 6.0 (GraphPad Software Inc., CA) software programs.

## Results

### Participants

We checked the eligibility of 65 patients who started glucocorticoid therapy at Chiba University Hospital between 1984 and 2014. Among them, 16 participants were not included in the study due to the loss of medical records or a lack of details regarding glucocorticoid therapy. Thirty-nine patients were included in the present study. Written informed consent was obtained from each of the study participants. No participants withdrew from the study.

### The patient characteristics

The characteristics of the participants are shown in Table 1. In the period between the initiation of glucocorticoid therapy and the evaluation of bone loss or osteoporosis, there were no participants with obesity, and none of the patients smoked or drank alcohol (data not shown). All of the participants had been hospitalized during the period (median, 85 days; interquartile range, 63 to 103), and the physical activity of the participants was limited during the period. We gave the participants guidance to help them maintain an appropriate level of physical activity after discharge from the hospital. We provided standard-nutrition meals in the hospital and educated the patients and their guardians on continuing to consume standard-nutrition meals after discharge from the hospital. Approximately 77% of the participants were treated with some kind of immunosuppressive drug (6 mizoribine, 2 cyclosporine, 5 tacrolimus, 9 intravenous cyclophosphamide, 4 mycophenolate mofetil, and 8 methotrexate, including multiple agents in some) together with glucocorticoids during the period. Two patients with systemic juvenile idiopathic arthritis received tocilizumab. Approximately 46% of the participants received alendronate therapy before the evaluation of osteoporosis. The median weekly dose per body surface area was 25.4 mg/m^2^ (interquartile range, 23.3 to 28.6). The participants treated with alendronate were not suffering from any adverse effects, including osteonecrosis of the jaw, before the evaluation of osteoporosis or before the enrollment (Table 1). No participants were missing data for any of these variables.

**Table 1:** Patient characteristics

Age at the enrollment (years, median [IQR])	22.5 [17.5 to 28.1]
Female gender	79.5%
Age at the onset of primary disease (years, median [IQR])	10.6 [8.1 to 13.0]
Primary diseases	
SLE	56.4%
sJIA	15.4%
Others	28.2%
Age at the evaluation of osteoporosis (years, median [IQR])	12.0 [9.4 to 14.3]
Age at the initiation of glucocorticoid therapy (years, median [IQR])	11.2 [8.2 to 13.2]
Body weight at the initiation of glucocorticoid therapy (kg, median [IQR])	36.8 [24.2 to 43.0]
Hospitalization during the study period	100%
Length of hospitalization during the study period (days, median [IQR])	85 [63 to 103]
Length of the period between at the initiation of glucocorticoid therapy and evaluation of osteoporosis (years, median [IQR])	0.9 [0.7 to 1.1]
Cumulative prednisolone-equivalent dose of glucocorticoids (mg, median [IQR])	12,043 [8790 to 14,213]
Cumulative prednisolone-equivalent dose of glucocorticoids per body weight per day (mg/kg/day, median [IQR])	1.2 [0.8 to 1.5]
Number of mPSLPT (median [IQR])	2 [1 to 2]
Cumulative prednisolone-equivalent dose of glucocorticoids except mPSLPT (mg, median [IQR])	5881 [4606 to 7073]
Cumulative prednisolone-equivalent dose of glucocorticoids per body weight per day except mPSLPT (mg/kg/day, median [IQR])	0.6 [0.5 to 0.8]
Use of immunosuppressive drugs^a^	76.9%
Use of tocilizumab	5.1%
Supplementation of vitamin D	38.5%
Supplementation of calcium	5.1%
Alendronate therapy	
before the enrollment	79.5%
Age at the initiation (years, median [IQR])	13.6 [10.0 to 15.5]
Osteonecrosis of the jaw	0%
Other adverse effects	0%
before the evaluation of osteoporosis	46.2%
Age at the initiation (years, median [IQR])	11.5 [8.0 to 14.1]
Osteonecrosis of the jaw	0%
Other adverse effects	0%
within 3 months after the initiation of glucocorticoid therapy	30.8%

*IQR* interquartile range, *SLE* systemic lupus erythematosus, *sJIA* systemic juvenile idiopathic arthritis, *mPSLPT* methylprednisolone pulse therapy; ^a^mizoribine, cyclosporine, tacrolimus, intravenous cyclophosphamide, mycophenolate mofetil, or methotrexate

### Outcomes

**Fig. 1:**
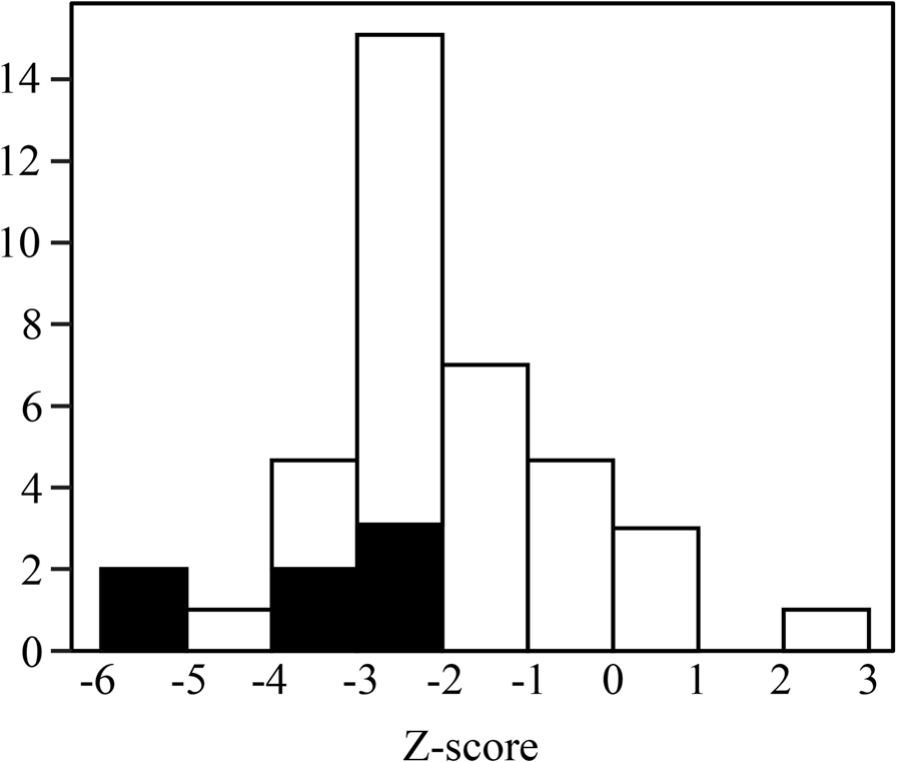
The Z-scores of the BMD of the spine (L2–4) in the whole study population. The black portion indicates the participants with a history of fracture

The distribution of the Z-scores of the LS BMD and the presence of a fracture history are shown in Fig. 1. Fifty-six percent of the participants had a LS BMD Z-score of ≤ − 2 (median, − 2.14; interquartile range, − 2.83 to − 1.29) and 17.9% had a fracture history. All of the patients with a fracture history had vertebral compression fractures; none had long bone fractures of the lower extremities or two or more long bone fractures of the upper extremities (Table 2). Participants with bone loss (wBL group) had significantly lower LS BMD Z-scores (*p* <  0.01) and the rate of patients with a fracture history was significantly higher in comparison to those without bone loss (w/oBL group) (*p* = 0.03) (Table 2).

**Table 2:** The characteristics and outcomes of the patients with and without bone loss

	wBL group (*N* = 23)	w/oBL group (*N* = 16)	*p*-value
Characteristics			
Female gender	82.6%	75%	0.69
Age at the onset of primary disease (years, median [IQR])	10.5 [6.8 to 12.9]	10.9 [9.1 to 13.0]	0.27
Primary disease			
SLE	60.9%	50.0%	0.53
sJIA	21.7%	6.3%	0.37
Others	17.4%	43.8%	0.15
Age at the evaluation of osteoporosis (years, median [IQR])	11.7 [8.0 to 14.6]	12.2 [10.9 to 13.7]	0.42
Age at the initiation of glucocorticoid therapy (years, median [IQR])	11.0 [7.3 to 14.0]	11.4 [10.0 to 13.2]	0.28
Body weight at the initiation of glucocorticoid therapy (kg, median [IQR])	33.0 [20.4 to 44.2]	37.1 [29.3 to 42.1]	0.51
Hospitalization during the study period	100%	100%	1.00
Length of hospitalization during the study period (days, median [IQR])	94 [68 to 110]	78 [56.5 to 88]	0.11
Length of the period between the initiation of glucocorticoid therapy and evaluation of osteoporosis (years, median [IQR])	0.9 [0.7 to 1.2]	0.8 [0.6 to 1.0]	0.14
Cumulative prednisolone-equivalent dose of glucocorticoids (mg, median [IQR])	11,300 [8146 to 15,260]	12,501 [9216 to 13,777]	0.83
Cumulative prednisolone-equivalent dose of glucocorticoids per body weight per day (mg/kg/day, median [IQR])	1.1 [0.8 to 1.5]	1.2 [0.6 to 1.5]	0.66
Number of mPSLPT (median [IQR])	2 [1 to 2]	2 [2 to 2]	0.75
Cumulative prednisolone-equivalent dose of glucocorticoids except mPSLPT (mg, median [IQR])	6222 [4353 to 8973]	5678 [4764 to 6637]	0.35
Cumulative prednisolone-equivalent dose of glucocorticoids per body weight per day except mPSLPT (mg/kg/day, median [IQR])	0.6 [0.5 to 0.8]	0.6 [0.4 to 0.7]	0.33
Use of immunosuppressive drugs^a^	82.6%	68.8%	0.44
Use of tocilizumab	8.7%	0%	0.50
Supplementation of vitamin D	43.5%	61.5%	0.52
Supplementation of calcium	0.0%	12.5%	0.16
Alendronate therapy			
before the evaluation of osteoporosis	34.7%	62.5%	0.11
within 3 months after the initiation of glucocorticoid therapy	13.0%	56.3%	< 0.01
Outcomes			
Z-score of L2–4 lumbar BMD (median [IQR])	−2.74 [−3.62 to −2.29]	−0.61 [−1.51 to 0.18]	< 0.01
Fracture history	30.4%	0%	0.03
Long bone fracture of the lower extremities	0%	0%	1.00
Vertebral compression fracture	30.4%	0%	0.03
Two or more long bone fractures of the upper extremities	0%	0%	1.00

*IQR* interquartile range, *SLE* systemic lupus erythematosus, *sJIA* systemic juvenile idiopathic arthritis, *mPSLPT* methylprednisolone pulse therapy, *wBL* group, participants with bone loss, *w/oBL* group, participants without bone loss; ^a^mizoribine, cyclosporine, tacrolimus, intravenous cyclophosphamide, mycophenolate mofetil, or methotrexate

### Risk factors for the development of bone loss and osteoporosis

There were no significant differences between the wBL and w/oBL groups in terms of gender, primary disease, age at the onset of primary disease, the age at the evaluation of osteoporosis, the use of immunosuppressive drugs, or the use of tocilizumab (Table 2). In addition, we did not find any difference between the groups with regard to the use of vitamin D, calcium supplementation, or the length of hospitalization in which the physical activity of the participants was required to be low. In terms of glucocorticoid use, there was no significant difference in the age at the start of glucocorticoid therapy, the length of glucocorticoid use, or the dose of glucocorticoids. The proportion of patients in the w/oBL group who received alendronate (62.5%) tended to be larger than that in the wBL group (34.7%). Moreover, the proportion of patients in the w/oBL group who received alendronate within 3 months after the start of glucocorticoid therapy (56.3%) was significantly greater than that in the wBL group (13.0%). As we expected, no participants who were treated with alendronate had a fracture history and the proportion of patients receiving alendronate among the participants without osteoporosis (58.3%) was significantly greater than that among the patients with osteoporosis (Additional file 1: Table S1). We did not find a significant difference in the proportion of patients who received alendronate within 3 months after the initiation of glucocorticoid therapy between the participants with and without osteoporosis (*p* = 0.08).

In the multivariate regression analysis (Table 3), factors other than “supplementation of calcium” and “alendronate therapy within 3 months after the start of glucocorticoid therapy” were excluded from the analysis in a step-wise manner. In this logistic regression analysis, only “alendronate therapy within 3 months after the start of glucocorticoid therapy” was significantly associated with the development of bone loss (odds ratio [OR], 0.08; 95% confidence interval [CI], 0.02–0.43; *p* <  0.01).

**Table 3:** The univariate and multivariate regression analyses of factors associated with bone loss

	Univariate analysis		Multivariate analysis	
	OR [95% CI]	*p*-value	OR [95% CI]	*p*-value
Female gender	1.58 [0.33–7.56]	0.56	Excluded	
Older age at the onset of primary disease (per 1 year)	0.86 [0.68–1.07]	0.18	Excluded	
Primary disease				
SLE	1.56 [0.43–5.65]	0.50	Excluded	
sJIA	4.17 [0.44–39.7]	0.21	Excluded	
Others	0.27 [0.06–1.17]	0.08	Excluded	
Older age at the evaluation of osteoporosis (per 1 year)	0.90 [0.73–1.11]	0.32	Excluded	
Older age at the initiation of glucocorticoid therapy (per 1 year)	0.88 [0.71–1.09]	0.24	Excluded	
Heavier body weight at the initiation of glucocorticoid therapy (per 1 kg)	0.97 [0.91–1.02]	0.22	Excluded	
Hospitalization during the study period	NC	NC	Excluded	
Longer length of hospitalization during the study period (per 1 day)	1.01 [1.00–1.03]	0.18	Excluded	
Longer length of the period between the initiation of glucocorticoid therapy and evaluation of osteoporosis (per 1 year)	8.86 [0.65–121.3]	0.10	Excluded	
Greater cumulative prednisolone-equivalent dose of glucocorticoids (per 1 mg)	1.00 [1.00–1.00]	0.83	Excluded	
Greater cumulative prednisolone-equivalent dose of glucocorticoids per body weight per day (per 1 mg/kg/day)	0.95 [0.31–2.89]	0.92	Excluded	
Greater number of mPSLPT (per time)	1.04 [0.59–1.83]	0.89	Excluded	
Greater cumulative prednisolone-equivalent dose of glucocorticoids except mPSLPT (per 1 mg)	1.00 [1.00–1.00]	0.14	Excluded	
Greater cumulative prednisolone-equivalent dose of glucocorticoids per body weight per day except mPSLPT (per 1 mg/kg/day)	5.07 [0.23–113.0]	0.31	Excluded	
Use of immunosuppressive drugs^a^	2.16 [0.48–9.77]	0.32	Excluded	
Use of tocilizumab	8,293,320 [0.00-NC]	0.99	Excluded	
Supplementation of vitamin D	1.69 [0.44–6.47]	0.44	Excluded	
Supplementation of calcium	0.12 [0.01–2.72]^b^	0.12	0.00 [0.00-NC]	0.99
Alendronate therapy				
by the evaluation of osteoporosis	0.32 [0.08–1.21]	0.09	Excluded	
within 3 months after the initiation of glucocorticoid therapy	0.12 [0.02–0.56]	< 0.01	0.08 [0.02–0.43]	< 0.01

*SLE* systemic lupus erythematosus, *sJIA* systemic juvenile idiopathic arthritis, *mPSLPT* methylprednisolone pulse therapy; ^a^mizoribine, cyclosporine, tacrolimus, intravenous cyclophosphamide, mycophenolate mofetil, or methotrexate; ^b^, adjusted relative risk; *NC* not calculated

To clarify the effects of the early administration of alendronate on the development of bone loss or osteoporosis, we performed subgroup analyses. As shown in Additional file 2: Table S2, the participants who received alendronate therapy had started glucocorticoids later in the study. The proportion of patients with systemic lupus erythematosus among the participants who received alendronate therapy (33.3%) was significantly smaller than that in the participants who did not receive alendronate therapy (76.2%). The primary diseases other than systemic lupus erythematosus and systemic juvenile idiopathic arthritis among the patients who received alendronate therapy included mixed connective tissue disease (*n* = 3), dermatomyositis (*n* = 3), and 3 Takayasu arteritis (*n* = 3). Thus, the bias in the primary diseases of the participants who received alendronate therapy, was smaller than that in the parent population.

Among the participants treated with alendronate, the length of the period between the initiation of glucocorticoid therapy and the initiation of alendronate therapy was significantly shorter in the patients without bone loss (*p* = 0.02) (Additional file 3: Table S3). The proportion of patients who received alendronate within 3 months after the initiation of glucocorticoid therapy among the patients without bone loss (90.0%) was significantly greater than that in the patients with bone loss (37.5%, *p* = 0.04). In contrast, the weekly dose per body surface area and the length of alendronate therapy did not differ markedly between the groups. Among the patients who received alendronate with 3 months after the initiation of glucocorticoid therapy, the presence of bone loss was significantly lower and the LS BMD Z-score was significantly higher in comparison to the participants who received alendronate more than 3 months after the initiation of glucocorticoid therapy (Additional file 4: Table S4). Due to absence of the participants with osteoporosis among the alendronate-treated participants, we could not evaluate the risk factors for the development of osteoporosis in the population.

## Discussion

In the present study, we demonstrated that the initiation of alendronate therapy within 3 months after the initiation of glucocorticoid therapy was a protective factor against the development of bone loss in the glucocorticoid-treated patients with childhood-onset rheumatic disease. This suggests that— similarly to adults [13]—the early use of bisphosphonates might have a preventive effect against the development of glucocorticoid-induced osteoporosis in children.

### Limitations

The present study was associated with some limitations. First, the study population was relatively small because childhood-onset rheumatic diseases are rare. In addition, the primary diseases of the participants were heterogeneous. As the inflammatory activity, which influences the development of bone loss and osteoporosis, differed between the primary diseases, a further study to evaluate the risk factors for each primary disease is needed. We were also unable to evaluate the impact of the disease activity of each primary disease due to the heterogeneity of the study population. However, we found no marked differences in the proportion of the participants who received concomitant immunosuppressive drugs or biologics, suggesting that reductions in the disease activity by immunosuppressive drugs or biologics may not have had a marked influence on the development of bone loss in the study population. Second, there might have been a referral filter bias because patients with a high risk of bone loss might have been more likely to be referred to (and treated in) our hospital. Further multi-center studies are needed to confirm the results. Finally, we did not evaluate the lifestyle habits that might have affected the bone loss [14], including physical activity and consumption of calcium-rich foods. However, the length of hospitalization, during which the physical activity of the participants was required to be low, did not significantly influence the development of bone loss or osteoporosis, suggesting that the reduced physical activity while in the hospital might not have strongly influenced the development of bone loss in our population. Regarding the consumption of calcium-rich foods, we provided standard-nutrition meals to patients in the hospital and educated the patients and their guardians on continuing to consume standard-nutrition meals after discharge. We therefore suspect that none of the patients had any calcium deficiency that might have caused bone loss.

### Interpretation

In our study, patients with childhood-onset rheumatic diseases who were treated with glucocorticoids had a higher prevalence of bone loss and vertebral fracture in comparison to the results reported from a national survey in Canada [15]. This is probably because the cumulative glucocorticoid dose of the patients in this study (mean, 10,184 mg/m^2^; SD, 4673 mg/m^2^; data not shown in Table 1) was greater than that of the patients in the Canadian study (mean, 6369 mg/m^2^; SD, 5146 mg/m^2^ [15]).

In terms of the analysis of risk factors for the development of bone loss and osteoporosis, we found that early alendronate therapy was the only protective factor against the development of bone loss. As shown in Additional file 3: Table S3 and Additional file 4: Table S4, even if we focused on alendronate-treated participants alone, the initiation of alendronate therapy within 3 months after the start of glucocorticoid therapy clearly associated with a decreased frequency of bone loss. Glucocorticoid-induced bone loss occurs early after the initiation of glucocorticoid therapy due to relatively higher bone resorption as a result of the suppression of bone formation by glucocorticoid treatment. In childhood, the larger imbalance of bone metabolism due to glucocorticoid treatment is particularly concerning because the bone turnover is relatively high in comparison to that in adulthood. Thus, early intervention to prevent bone loss in glucocorticoid-treated children should be discussed.

In contrast, we did not find any risk factors associated with the development of osteoporosis; this was probably due to the small number of participants who developed osteoporosis in our study population.

Although the fracture risk in glucocorticoid-treated children with rheumatic disease is unknown at the time of writing, a preventive approach should be considered for children undergoing glucocorticoid therapy because their treatment is likely to continue until adulthood.

### Generalizability

The rate of vitamin D and calcium supplementation, which is recommended for glucocorticoid-treated children [5], was low in our study because they were not administered in most cases in order to avoid the development of urinary stones. This might affect the generalizability of our findings in terms of the impact of these factors on the development of bone loss.

## Conclusions

In conclusion, our study found that early use of alendronate might have a preventive effect against the development of glucocorticoid-induced osteoporosis in children. Further interventional studies are needed to clarify whether the early administration of alendronate reduces the long-term fracture risk.

## Additional files

Additional file 1: **Table S1.** A comparison of the characteristics and outcomes of patients with and without osteoporosis. (DOCX 25 kb)

Additional file 2: **Table S2.** The characteristics and outcomes of participants treated with and without alendronate. (DOCX 25 kb)

Additional file 3: **Table S3.** The characteristics and outcomes of alendronate-treated patients with and without bone loss. (DOCX 26 kb)

Additional file 4: **Table S4.** The outcomes of the alendronate-treated participants who started alendronate within 3 months of the initiation of glucocorticoid therapy and more than 3 months after the initiation of glucocorticoid therapy. (DOCX 20 kb)

## Abbreviations

BMD: Bone mineral density
CI: Confidence interval
IQR: Interquartile range
mPSLPT: methylprednisolone pulse therapy
NC: Not calculated
OR: Odds ratio
SD: Standard deviation
sJIA: Systemic juvenile idiopathic arthritis
SLE: Systemic lupus erythematosus
w/oBL group: Participants without bone loss
wBL group: Participants with bone loss
