# Supplementary material for: Early use of alendronate as a protective factor against the development of glucocorticoid-induced bone loss in childhood-onset rheumatic diseases: a cross-sectional study
Source: Pediatr Rheumatol Online J. 2018 Jun 18;16:36. doi: 10.1186/s12969-018-0258-5 (PMC6006935; doi:10.1186/s12969-018-0258-5)
Supplement: Supplementary file 1 — Table S1. A comparison of the characteristics and outcomes of patients with and without osteoporosis. (DOCX 25 kb) [file 12969_2018_258_MOESM1_ESM.docx]

**Additional file 1: Table S1. A comparison of the characteristics and outcomes of patients with and without osteoporosis**

|  | | | Osteoporosis (+) (N = 7) | Osteoporosis (-) (N = 32) | p-value |
| --- | --- | --- | --- | --- | --- |
| **Characteristics** | | |  |  |  |
| Female gender | | | 71.4% | 28.6% | 0.62 |
| Age at the onset of primary disease (years, median [IQR]) | | | 10.3 [5.5 to 10.9] | 10.8 [8.2 to 13.0] | 0.41 |
| Primary disease | | |  |  |  |
|  | | SLE | 57.1% | 56.3% | 1.00 |
|  | | sJIA | 28.6% | 12.5% | 0.29 |
|  | | Others | 14.3% | 31.3% | 0.65 |
| Age at the evaluation of osteoporosis (years, median [IQR]) | | | 11.7 [6.4 to 15.4] | 12.1 [9.4 to 14.3] | 0.63 |
| Age at the initiation of glucocorticoid therapy (years, median [IQR]) | | | 11.0 [5.6 to 14.6] | 11.3 [8.3 to 13.2] | 0.69 |
| Body weight at the initiation of glucocorticoid therapy (kg, median [IQR]) | | | 42.4 [22.4 to 46.3] | 33.0 [24.6 to 42.9] | 0.36 |
| Hospitalization during the study period | | | 100% | 100% | 1.00 |
| Length of hospitalization during the study period (days, median [IQR]) | | | 110 [63 to 237] | 83 [62.75 to 99] | 0.19 |
| Length of the period between the initiation of glucocorticoid therapy and the evaluation of osteoporosis (years, median [IQR]) | | | 0.8 [0.7 to 1.2] | 0.9 [0.6 to 1.1] | 0.94 |
| Cumulative prednisolone-equivalent dose of glucocorticoids (mg, median [IQR]) | | | 11923 [8973 to 21025] | 12061 [8307 to 13777] | 0.69 |
| Cumulative prednisolone-equivalent dose of glucocorticoids per body weight per day (mg/kg/day, median [IQR]) | | | 1.1 [0.5 to 1.7] | 1.2 [0.8 to 1.5] | 0.91 |
| Number of mPSLPT (median [IQR]) | | | 2 [0 to 4] | 2 [1 to 2] | 0.95 |
| Cumulative prednisolone-equivalent dose of glucocorticoids except mPSLPT (mg, median [IQR]) | | | 7495 [5736 to 9455] | 5722 [4416 to 6637] | 0.09 |
| Cumulative prednisolone-equivalent dose of glucocorticoids per body weight per day except mPSLPT (mg/kg/day, median [IQR]) | | | 0.5 [0.4 to 0.9] | 0.6 [0.5 to 0.7] | 0.69 |
| Use of immunosuppressive drugs* | | | 71.4% | 78.1% | 0.65 |
| Use of tocilizumab | | | 0.0% | 6.25% | 1.00 |
| Supplementation of vitamin D | | | 42.9% | 37.5% | 1.00 |
| Supplementation of calcium | | | 0.0% | 6.3% | 1.00 |
| Alendronate therapy | | |  |  |  |
|  | before the evaluation of osteoporosis | | 0.0% | 56.3% | <0.01 |
|  | within 3 months after the initiation of glucocorticoid therapy | | 0.0% | 37.5% | 0.08 |
| **Outcomes** | | |  |  |  |
| Z-score of L2-4 lumbar BMD (median [IQR]) | | | -3.13 [-5.14 to -2.14] | -1.98 [-2.70 to -0.45] | 0.01 |
| Fracture history | | | 100.0% | 0% | <0.01 |
|  | Long bone fracture of the lower extremities | | 0% | 0% | 1.00 |
|  | Vertebral compression fracture | | 100.0% | 0% | <0.01 |
|  | Two or more long bone fractures of the upper extremities | | 0% | 0% | 1.00 |

SLE, systemic lupus erythematosus; sJIA, systemic juvenile idiopathic arthritis; IQR, interquartile range; mPSLPT, methylprednisolone pulse therapy; *mizoribine, cyclosporine, tacrolimus, intravenous cyclophosphamide, mycophenolate mofetil, or methotrexate
