# Supplementary material for: Early use of alendronate as a protective factor against the development of glucocorticoid-induced bone loss in childhood-onset rheumatic diseases: a cross-sectional study
Source: Pediatr Rheumatol Online J. 2018 Jun 18;16:36. doi: 10.1186/s12969-018-0258-5 (PMC6006935; doi:10.1186/s12969-018-0258-5)
Supplement: Supplementary file 2 — Table S2. The characteristics and outcomes of participants treated with and without alendronate. (DOCX 25 kb) [file 12969_2018_258_MOESM2_ESM.docx]

**Additional file 2: Table S2. The characteristics and outcomes of participants treated with and without alendronate**

|  | | | Alendronate therapy (+) (N = 18) | Alendronate therapy (-) (N = 21) | p-value |
| --- | --- | --- | --- | --- | --- |
| **Characteristics** | | |  |  |  |
| Female gender | | | 88.9% | 71.4% | 0.25 |
| Age at the onset of primary disease (years, median [IQR]) | | | 10.9 [7.8 to 13.5] | 10.6 [8.3 to 11.8] | 0.42 |
| Primary disease | | |  |  |  |
|  | | SLE | 33.3% | 76.2% | 0.01 |
|  | | sJIA | 16.7% | 14.3% | 1.00 |
|  | | Others | 50.0% | 9.5% | 0.01 |
| Age at the evaluation of osteoporosis (years, median [IQR]) | | | 12.2 [8.7 to 14.7] | 12.0 [9.5 to 13.8] | 0.67 |
| Year at the initiation of glucocorticoid therapy (median [IQR]) | | | 2012 [2009 to 2013] | 2002 [1999 to 2004] | <0.01 |
| Age at the initiation of glucocorticoid therapy (years, median [IQR]) | | | 11.4 [7.9 to 14.0] | 11.0 [8.4 to 12.8] | 0.61 |
| Body weight at the initiation of glucocorticoid therapy (kg, median [IQR]) | | | 34.9 [24.7 to 44.4] | 37.3 [24.1 to 43.0] | 0.87 |
| Hospitalization during the study period | | | 100% | 100% | 1.00 |
| Length of hospitalization during the study period (days, median [IQR]) | | | 79.5 [57.5 to 94.25] | 88 [69 to 139.5] | 0.15 |
| Length of the period between the initiation of glucocorticoid therapy and the evaluation of osteoporosis (years, median [IQR]) | | | 0.9 [0.6 to 1.0] | 0.8 [0.6 to 1.2] | 0.93 |
| Cumulative prednisolone-equivalent dose of glucocorticoids (mg, median [IQR]) | | | 12061 [8090 to 14146] | 11923 [9141 to 14654] | 0.98 |
| Cumulative prednisolone-equivalent dose of glucocorticoids per body weight per day (mg/kg/day, median [IQR]) | | | 1.2 [0.8 to 1.5] | 1.2 [0.8 to 1.6] | 0.91 |
| Number of mPSLPT (median [IQR]) | | | 2 [2 to 2] | 2 [0 to 2] | 0.44 |
| Cumulative prednisolone-equivalent dose of glucocorticoids except mPSLPT (mg, median [IQR]) | | | 4943 [4267 to 6469] | 6246 [5565 to 8855] | 0.07 |
| Cumulative prednisolone-equivalent dose of glucocorticoids per body weight per day except mPSLPT (mg/kg/day, median [IQR]) | | | 0.5 [0.4 to 0.6] | 0.7 [0.5 to 0.8] | 0.16 |
| Use of immunosuppressive drugs* | | | 88.9% | 66.7% | 0.14 |
| Use of tocilizumab | | | 11.1% | 0% | 0.21 |
| Supplementation of vitamin D | | | 44.4% | 33.3% | 0.53 |
| Supplementation of calcium | | | 5.6% | 4.8% | 1.00 |
| Alendronate therapy | | |  |  |  |
|  | within 3 months of the initiation of glucocorticoid therapy | | 66.7% | 0% | <0.01 |
| **Outcomes** | | |  |  |  |
| Z-score of L2-4 lumbar BMD (median [IQR]) | | | -1.87 [-2.41 to -0.14] | -2.57 [-3.44 to 1.63] | 0.03 |
| Fracture history | | | 0% | 33.3% | <0.01 |
| Bone loss | | | 44.4% | 71.4% | 0.11 |
| Osteoporosis | | | 0% | 33.3% | <0.01 |

SLE, systemic lupus erythematosus; sJIA, systemic juvenile idiopathic arthritis; IQR, interquartile range; mPSLPT, methylprednisolone pulse therapy; *mizoribine, cyclosporine, tacrolimus, intravenous cyclophosphamide, mycophenolate mofetil, or methotrexate
