# Supplementary material for: Early use of alendronate as a protective factor against the development of glucocorticoid-induced bone loss in childhood-onset rheumatic diseases: a cross-sectional study
Source: Pediatr Rheumatol Online J. 2018 Jun 18;16:36. doi: 10.1186/s12969-018-0258-5 (PMC6006935; doi:10.1186/s12969-018-0258-5)
Supplement: Supplementary file 3 — Table S3. The characteristics and outcomes of alendronate-treated patients with and without bone loss. (DOCX 26 kb) [file 12969_2018_258_MOESM3_ESM.docx]

**Additional file 3: Table S3. The characteristics and outcomes of alendronate-treated patients with and without bone loss**

|  | | | Bone loss (+) (N = 8) | Bone loss (-) (N = 10) | p-value |
| --- | --- | --- | --- | --- | --- |
| **Characteristics** | | |  |  |  |
| Female gender | | | 87.5% | 90.0% | 1.00 |
| Age at the onset of primary disease (years, median [IQR]) | | | 8.8 [5.5 to 13.3] | 12.1 [10.1 to 14.1] | 0.11 |
| Primary disease | | |  |  |  |
|  | | SLE | 37.5% | 30.0% | 1.00 |
|  | | sJIA | 25.0% | 10.0% | 0.56 |
|  | | Others | 37.5% | 60.0% | 0.64 |
| Age at the evaluation of osteoporosis (years, median [IQR]) | | | 9.9 [6.6 to 14.5] | 13.0 [11.1 to 15.1] | 0.25 |
| Age at the initiation of glucocorticoid therapy (years, median [IQR]) | | | 9.0 [5.8 to 13.7] | 12.3 [10.2 to 14.2] | 0.18 |
| Body weight at the initiation of glucocorticoid therapy (kg, median [IQR]) | | | 25.8 [17.4 to 43.7] | 38.7 [31.7 to 47.8] | 0.13 |
| Hospitalization during the study period | | | 100% | 100% | 1.00 |
| Length of hospitalization during the study period (days, median [IQR]) | | | 89.5 [65.75 to 101] | 77 [50.75 to 86.5] | 0.25 |
| Length of the period between the initiation of glucocorticoid therapy and the evaluation of osteoporosis (years, median [IQR]) | | | 0.9 [0.7 to 1.2] | 0.8 [0.6 to 1.0] | 0.37 |
| Cumulative prednisolone-equivalent dose of glucocorticoids (mg, median [IQR]) | | | 11689 [7979 to 14833] | 12065 [7961 to 14833] | 0.72 |
| Cumulative prednisolone-equivalent dose of glucocorticoids per body weight per day (mg/kg/day, median [IQR]) | | | 1.1 [0.8 to 2.0] | 1.2 [0.5 to 1.4] | 0.48 |
| Number of mPSLPT (median [IQR]) | | | 2 [1 to 2] | 2 [2 to 2] | 0.87 |
| Cumulative prednisolone-equivalent dose of glucocorticoids except mPSLPT (mg, median [IQR]) | | | 4772 [4096 to 6236] | 5211 [4506 to 6744] | 0.59 |
| Cumulative prednisolone-equivalent dose of glucocorticoids per body weight per day except mPSLPT (mg/kg/day, median [IQR]) | | | 0.6 [0.5 to 0.8] | 0.5 [0.4 to 0.6] | 0.25 |
| Use of immunosuppressive drugs* | | | 100.0% | 80.0% | 0.48 |
| Use of tocilizumab | | | 25.0% | 0.0% | 0.18 |
| Supplementation of vitamin D | | | 50.0% | 40.0% | 1.00 |
| Supplementation of calcium | | | 0.0% | 10.0% | 1.00 |
| Alendronate therapy | | |  |  |  |
|  | Length of the period between the initiation of glucocorticoid therapy and the initiation of alendronate therapy  (years, median [IQR]) | | 0.3 [0.1 to 0.4] | 0.1 [0.0 to 0.1] | 0.02 |
|  | within 3 months after the initiation of glucocorticoid therapy | | 37.5% | 90.0% | 0.04 |
|  | Age at the initiation of alendronate therapy  (years, median [IQR]) | | 9.3 [5.9 to 14.0] | 12.3 [10.3 to 14.4] | 0.25 |
|  | Weekly dose per body surface area (mg/m^2^, median [IQR]) | | 24.6 [21.7 to 28.5] | 25.9 [24.4 to 29.0] | 0.37 |
|  | Length of alendronate therapy (years, median [IQR]) | | 0.5 [0.4 to 0.9] | 0.8 [0.5 to 0.9] | 0.50 |
| **Outcomes** | | |  |  |  |
| Z-score of L2-4 lumbar BMD (median [IQR]) | | | -2.42 [-2.91 to -2.25] | -0.55 [-2.91 to 0.51] | <0.01 |
| Fracture history | | | 0% | 0% | 1.00 |

SLE, systemic lupus erythematosus; sJIA, systemic juvenile idiopathic arthritis; IQR, interquartile range; mPSLPT, methylprednisolone pulse therapy; *mizoribine, cyclosporine, tacrolimus, intravenous cyclophosphamide, mycophenolate mofetil, or methotrexate
