# Supplementary material for: Early use of alendronate as a protective factor against the development of glucocorticoid-induced bone loss in childhood-onset rheumatic diseases: a cross-sectional study
Source: Pediatr Rheumatol Online J. 2018 Jun 18;16:36. doi: 10.1186/s12969-018-0258-5 (PMC6006935; doi:10.1186/s12969-018-0258-5)
Supplement: Supplementary file 4 — Table S4. The outcomes of the alendronate-treated participants who started alendronate within 3 months of the initiation of glucocorticoid therapy and more than 3 months after the initiation of glucocorticoid therapy. (DOCX 20 kb) [file 12969_2018_258_MOESM4_ESM.docx]

**Additional file 4: Table S4.** **The outcomes of the alendronate-treated participants who started alendronate within 3 months of the initiation of glucocorticoid therapy and more than 3 months after the initiation of glucocorticoid therapy**

|  | Within 3 months (N = 12) | After 3 months (N = 6) | p-value |
| --- | --- | --- | --- |
| **Outcomes** |  |  |  |
| Bone loss | 25% | 83% | 0.04 |
| Osteoporosis | 0% | 0% | 1.00 |
| Z-score of L2-4 lumbar BMD (median [IQR]) | -1.14 [-2.15 to 0.32] | -2.37 [-2.99 to -1.99] | 0.02 |
| Fracture history | 0% | 0% | 1.00 |

IQR, interquartile range
